# Longitudinal patterns of lifestyle risk behaviours among UK adults with established cardiovascular disease: a latent transition analysis

**DOI:** 10.3389/fcvm.2023.1116905

**Published:** 2023-09-04

**Authors:** Teketo Kassaw Tegegne, Sheikh Mohammed Shariful Islam, Ralph Maddison

**Affiliations:** Institute for Physical Activity and Nutrition, Deakin University, Geelong, VIC, Australia

**Keywords:** cardiovascular disease, lifestyle risk behaviour, latent transition analysis, UK Biobank, lifestyle clustering

## Abstract

**Background:**

People with cardiovascular disease (CVD) need to engage in healthy lifestyle behaviours. However, there is a gap in identifying longitudinal patterns of change in lifestyle behaviours among people with CVD. This study aimed to identify clustering of lifestyle risk behaviours and their 4 ± year changes among UK adults with CVD, and to determine the associated factors.

**Methods:**

We used the UK Biobank data collected at two time points (2006–2010/baseline data = T_0_ and 2014+/third visit data = T_4_). Six key lifestyle risk behaviours were assessed: smoking, high alcohol intake, poor fruit and vegetable consumption, physical inactivity, poor sleep balance (<7 or >8 h/night) and prolonged sitting. A random intercept latent transition analysis was performed to identify patterns of lifestyle risk behaviours at T_0_ and their changes from T_0_ to T_4_.

**Results:**

We included 5,304 participants with CVD whose data on lifestyle risk behaviours were collected at two-time points. Alcohol intake and current smoking were 75.7% and 5.4% at baseline, respectively, and 67.4% and 3.0% at follow-up. Three latent classes emerged: Latent class (LC) 1—“high alcohol intake, poor sleep balance and poor fruit and vegetable intake”, LC2—“high alcohol intake and poor fruit and vegetable intake”, and LC3—“high alcohol intake”. Most adults remained in the same LC over the 4 + years (range: 83.9%–100.0%). After 4 + years, 3.5% from LC3 and 10.4% from LC2 at baseline moved into LC1. The odds of transitioning to LC2 relative to staying in LC1 and LC3 were 2.22 and 4.13 times higher for males than for females, respectively. A single-year increase in participants' age was associated with a 1.16 times increase in the odds of moving to LC1 relative to staying in LC2.

**Conclusion:**

People with CVD did not show improvement in lifestyle risk behaviours, and interventions targeting multiple lifestyle risk behaviours are needed to improve CVD.

## Background

Non-communicable diseases (NCDs) are the leading causes of death and disability worldwide (1), largely due to modifiable lifestyle risk behaviours such as tobacco use, alcohol consumption, physical inactivity, and unhealthy diet (2). Cardiovascular disease (CVD) remains the leading cause of disease burden globally (3). Modifying lifestyle risk behaviours could benefit individuals (e.g., improved quality of life (4) and well-being (5)), healthcare [e.g., reduced expenditure (6)], and society [e.g., increased productivity (7)].

Previous studies have shown adults often engage in multiple lifestyle risk behaviours. Prevalence of multiple risk behaviours reported among adults was 68% in England (8), 59% in Brazil (9), 55% in the Netherlands (10), 53.4% in Europe (11) and 52% in the United States of America (USA) (12). These lifestyle risk behaviours also tend to cluster or co-occur together within individuals (8, 13, 14). For example, smoking, unhealthy diet, physical inactivity, and excessive alcohol consumption cluster within certain people (15, 16). Those behaviours are more common among men, younger people, those with low socio-economic status and level of education (10, 13, 17–19).

Multiple lifestyle risk behaviours have synergetic detrimental health effects (20–23). A recent publication using the UK Biobank showed lifestyle risk behaviours were clustered within individuals and significantly increased CVD risk (24). A systematic review reported that people with multiple lifestyle risk behaviours were more likely to experience an incident CVD event, die from CVD, or any cause (25). To prevent CVD, the 2019 American Heart Association (AHA) guideline recommended people practice a healthy lifestyle over time (26). Those recommendations are consistent for people with an existing diagnosis of CVD (27–29). Currently, there is an evidence gap regarding how lifestyle risk factors change over time. Latent transition analysis (LTA) is a useful approach to identify clusters/latent classes of lifestyle risk behaviours in a heterogeneous population and their changes over time (30). LTA is an extension of latent class analysis to longitudinal data to estimate the probabilities of transitions among behaviour patterns over time (31).

This study aimed to identify clustering of lifestyle risk behaviours and their 4 ± year changes among UK adults with CVD and identify the factors associated with these changes. We focused on six lifestyle risk behaviours: smoking, high alcohol intake, poor fruit and vegetable consumption, physical inactivity, poor sleep balance, and prolonged sitting as these are the main modifiable causes of morbidity and mortality.

## Methods

UK Biobank has ethics approval from the North West Multi-centre Research Ethics Committee (32), and researchers do not require separate ethics applications to use this data. All participants provided written informed consent. This study followed the Strengthening the Reporting of Observational Studies in Epidemiology (STROBE) reporting guideline (33).

### Study population

Data from the United Kingdom (UK) Biobank study were used for this study. Details of the UK Biobank study design and population are described elsewhere (34, 35). Socio-demographic characteristics, lifestyle behaviours, and other health-related data were collected using the baseline questionnaire, interviews, and physical measurement (35). For this study, we used the UK Biobank data collected at two time points (2006–2010/baseline data = T0 and 2014+/third visit data = T4).

### Cardiovascular disease

**Figure 1:**
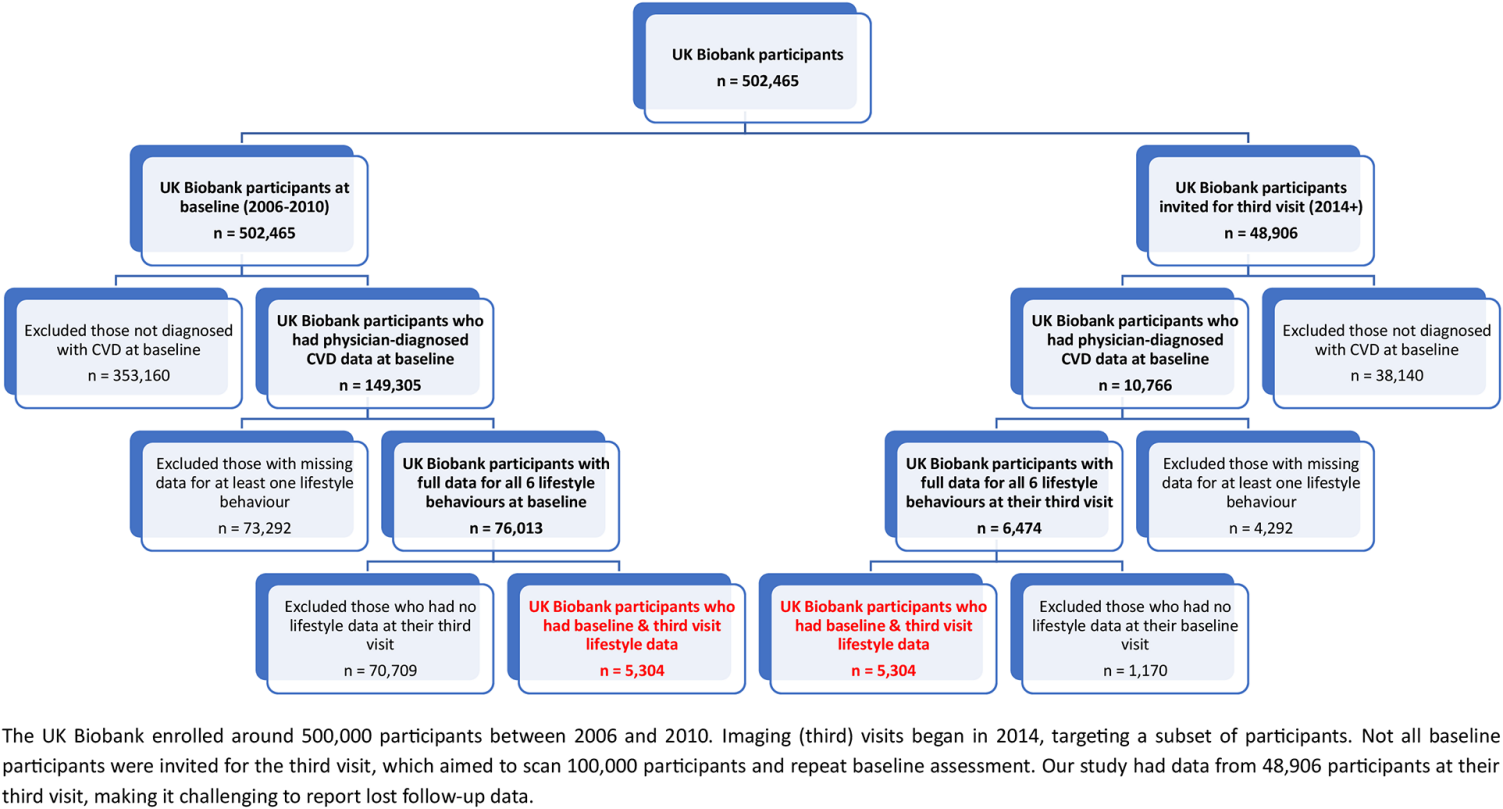
Illustrates the flowchart outlining the inclusion of participants with CVD. The final groups are highlighted in red.

The UK Biobank collected self-reported medical data, including physician-diagnosed CVD. To determine participants' CVD status in our study, we used doctor-diagnosed heart and vascular issues like heart attacks, angina, and strokes (Field ID = 6150). In this study, participants with at least one of these conditions were categorized as having CVD. Those without a doctor-diagnosed CVD at baseline, had missing lifestyle or covariate data were excluded (Figure 1).

### Lifestyle behaviour measures

#### Physical activity

For physical activity, UK Biobank data on time spent on moderate and vigorous activity was added and converted to a metabolic equivalent of task (MET) score. Based on the 2019 AHA guideline (26), people were classified as active if they had ≥750 MET min/week or inactive (<750 MET min/week).

#### Fruit and vegetable intake

To determine daily fruit and vegetable consumption, data on fresh fruit, dried fruit, salad/raw vegetable, and cooked vegetable were converted into portions. Consistent with the NHS guidelines (36, 37), participants who had eaten fruits and vegetables at least 5 portions per day were considered to have adequate fruit and vegetable intake and those with <5 portions per day were classified as poor.

#### Alcohol consumption

Since alcoholic drinks differ in the amount of alcohol content, each drink was converted into equivalent standard units, where 1 unit contains 10 ml of ethyl alcohol (38). Total weekly units of alcohol consumption were calculated by adding the units of beer, wine, and spirits. Based on the NHS guidelines (38), participants were grouped as low-risk drinkers (≤14 units per week) or high-risk drinkers (*>*14 units per week).

#### Smoking

To determine smoking status, participants were asked, “Do you smoke tobacco now?”. Response options were “Yes, on most or all days”, “Only occasionally” and “No”. Responses were binary coded so that those who responded “yes” or “smoke occasionally” were coded as 1, current smoker, while those who responded as “no” were coded as 0, not a current smoker.

#### Prolonged sitting

To determine total sitting time, hours spent watching television, using the computer, and driving during a typical day were combined. Based on the total time spent on sitting, participants were categorized as low-risk sitting (<8 h/day) or prolonged sitting (≥8 h/day) (39, 40). This was based on the evidence of greater mortality risk for each increased sitting time category compared with <8 h/day (39, 40).

#### Sleep duration

For sleep duration, the UK Biobank asked participants “*About how many hours sleep do you get in every 24 h? (please include naps)”.* Sleep duration was split using predefined thresholds from the literature; <7 h, 7–8 h and >8 h (41). Based on these cut points, participants were grouped as having “poor sleep balance” (<7 or >8 h/night) and “good sleep balance” (7–8 h/night).

### Socio-demographic variables

Socio-demographic characteristics (age and gender), and Townsend deprivation index (TSDI) were covariates included in the latent transition analysis (LTA) model. TSDI was used to measure participants' deprivation (42). The index combines information on housing, employment, car availability and social class, with higher values indicating greater deprivation (42).

### Statistical analysis

Data on baseline characteristics, lifestyle behaviours and CVD status during the initial assessment and third visits were extracted. A random intercept LTA (RI-LTA) (43) was performed to identify patterns of lifestyle risk behaviours at T_0_ and their changes from T_0_ to T_4_ using the Mplus version 8.8 software (44). To select the number of latent classes that best fit the data, first, a two-class latent model was fitted and successively increased the number of classes by one, up to a six-class latent model. Model evaluation was made using the Bayesian Information Criterion (BIC) and Akaike information criterion (AIC) (45). Model selection was made based on statistical criteria (with lower AIC and BIC) in conjunction with interpretability of the estimated latent classes (45). Based on these criteria, three latent classes were selected. The three latent classes were labelled as LC1: “high-risk drinker, poor sleep balance and poor fruit and vegetable intake”, LC2: “high-risk drinker and poor fruit and vegetable intake”, and LC3: “high-risk drinker”. To ensure that lifestyle risk behaviours have the same meaning at T_0_ and T_4_, measurement invariance was considered in the analysis (30).

A multivariable multinomial logistic regression model was run to identify factors associated with lifestyle risk behaviours at T_0_ and their changes from T_0_ to T_4_. We followed a two-step RI-LTA estimation procedure (43). The first step was a simple RI-LTA estimation without covariate. In the 2-step estimation, parameters were fixed to SVALUES obtained in the first step and all covariates were included in the model. The included covariates were age at recruitment, gender, and TSDI. We used the diagonal–stayer class as a reference to easily understand transitions instead of using the last LC as a reference. Statistical significance was determined at a *p*-value of 0.05, and outcomes were reported as odds ratio (OR) with 95% confidence interval (CI).

## Results

### Population characteristics

In this analysis, we included 5,304 participants with CVD whose data on lifestyle risk behaviours were collected at baseline (2006–2010) and during the third visit (2014 +). The mean age of participants at baseline was 58.4 (standard deviation ±6.6) years. Most participants were male (67.1%). At baseline, the prevalence of physician-diagnosed hypertension and diabetes mellitus were 73.47% and 5.15%, respectively.

**Figure 2:**
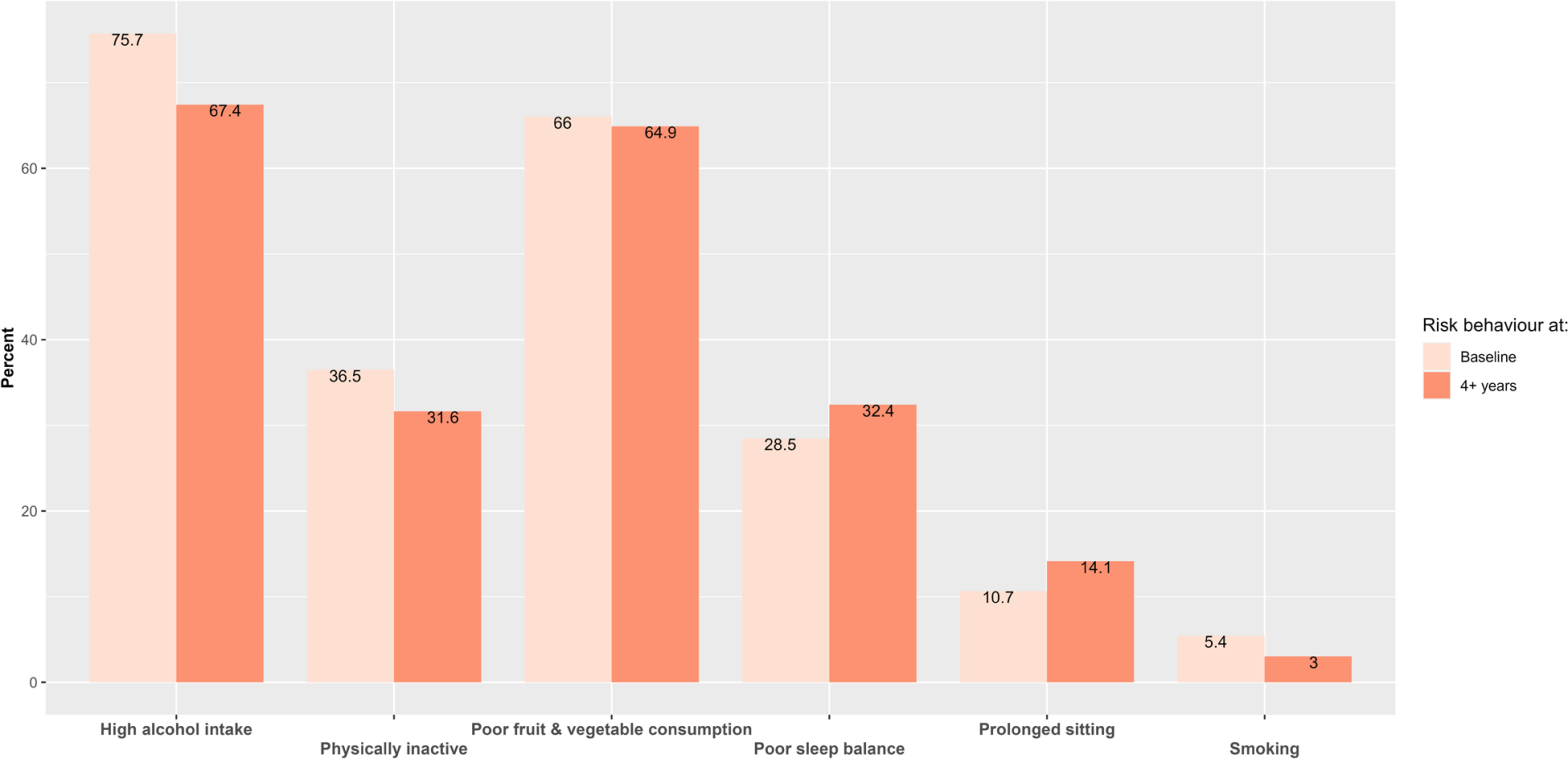
Lifestyle risk behaviours at baseline and after 4 + years.

Baseline alcohol intake was very high (75.7%), while active smokers within the last year comprised 5.45% of individuals (Figure 2). Those who had quit smoking after consuming at least 100 cigarettes accounted for 59.20%, and never-smokers constituted 35.35%. In terms of sleep, 28.47% of participants had poor sleep balance at baseline; 21.36% slept fewer than 7 h per night. This pattern remained consistent across genders: 20.9% of men and 22.3% of women slept less than 7 h, while 7.25% of men and 6.82% of women slept more than 8 h. Except for sitting and sleep, UK adults showed a slight decrease in engaging in lifestyle risk behaviours ranging from 1.1% (poor fruit and vegetable consumption) to 9.3% (high alcohol intake) over time. There was a slight increase in the prevalence of prolonged sitting (3.4% increase) and poor sleep balance (3.9% increase) from baseline to after 4 + years (Figure 2).

### Latent classes of lifestyle risk behaviours

A model with three latent classes were selected; LC1: “high alcohol intake, poor sleep balance and poor fruit and vegetable intake”, LC2: “high alcohol intake and poor fruit and vegetable intake”, and LC3: “high alcohol intake” (see Table 1). Participants in LC1 had a high probability of high alcohol intake (58.2%), poor sleep balance (75.3%) and poor fruit and vegetable intake (76.3%). LC2 was characterised by high alcohol intake (78.3%) and poor fruit and vegetable consumption (84.7%). Participants in LC3 had a high alcohol intake (72.8%).

**Table 1:** Lifestyle risk behaviour probability profiles.

Risk behaviours	Latent class		
	LC1	LC2	LC3
Physical inactivity	0.40	0.36	0.24
High alcohol intake	**0**.**58**	**0**.**78**	**0**.**73**
Prolonged sitting	0.17	0.12	0.10
Poor sleep balance	**0**.**75**	0.11	0.25
Poor fruit and vegetable intake	**0**.**76**	**0**.**85**	0.15
Smoking	0.03	0.07	0.01

Probability values ≥ 0.50 are in bold.

### Lifestyle risk behaviour transitions over time

Overall, adults remained mostly in the same LC from T_0_ to T_4_ (range: 83.9%–100.0%). LC2 had the highest prevalence at both time points, with 54.47% at T_0_ and 46.25% at T_4_ (Table 2). All participants in LC1 stayed in the same LC over time. Smaller changes were observed from LC2 to LC1 (10.4%) and LC3 (5.7%), from LC3 to LC1 (3.5%) (Table 2).

**Table 2:** Prevalence of lifestyle risk behaviours and their changes from T_0_ to T_4_.

	Latent class 1	Latent class 2	Latent class 3
Prevalence (%) of lifestyle risk behaviours at:			
T_0_	1,149 (21.7)	2,889 (54.5)	1,266 (23.9)
T_4_	1,480 (27.9)	2,453 (46.3)	1,371 (25.9)
Latent transition probabilities (%) (rows for T_0_, columns for T_4_)			
LC1	**1,149** (100.0*)	0.0	0.0
LC2	300 (10.4)	**2,424** (83.9*)	165 (5.7)
LC3	44 (3.5)	0.0	**1,222** (96.5*)

* Latent transition probabilities correspond to memberships in the same lifestyle risk behaviours at both time points.

Probability values ≥ 0.50 are in bold.

### Factors associated with lifestyle risk behaviour changes from T_0_ to T_4_

The odds of transitioning to LC2 relative to staying in LC1 was 2.22 times higher for males than for females [OR = 2.22 (1.16, 4.25)]. Similarly, the odds of moving to LC2 relative to staying in LC3 was 4.128 times higher for males than for females [OR = 4.13 (1.40, 12.19)]. A single-year increase in the age of participants at baseline was associated with a 1.16 times increase in the odds of transitioning to LC1 relative to staying in LC2 [OR = 1.16 (1.04, 1.29)]. Social deprivation measured in TSDI did not show a statistically significant effect on the transition probability of risk lifestyle behaviour profiles (see Table 3).

**Table 3:** Factors associated with lifestyle risk behaviour changes from T_0_ to T_4_.

Baseline (T_0_)	4 + years (T_4_)		
	OR with 95% CI		
	Latent class 1	Latent class 2	Latent class 3
Latent transition odds ratios and 95% confidence intervals for T_0_ to T_4_ (rows for T_0_, columns for T_4_)			
Gender (Male = 1)			
LC1	1.00 (1.00, 1.00)	**2.22** (**1.16, 4.25)**	0.54 (0.15, 1.95)
LC2	**0.45** (**0.24, 0.87)**	1.00 (1.00, 1.00)	**0.24** (**0.08, 0.72)**
LC3	1.86 (0.51, 6.84)	**4.13** (**1.40, 12.19)**	1.00 (1.00, 1.00)
Age at recruitment			
LC1	1.00 (1.00, 1.00)	**0.86** (**0.78, 0.96)**	0.92 (0.82, 1.03)
LC2	**1.16** (**1.04, 1.27)**	1.00 (1.00, 1.00)	1.07 (0.98, 1.16)
LC3	1.09 (0.98, 1.22)	0.95 (0.86, 1.02)	1.00 (1.00, 1.00)
TSDI at recruitment			
LC1	1.00 (1.00, 1.00)	0.95 (0.85, 1.07)	0.96 (0.83, 1.14)
LC2	1.05 (0.93, 1.18)	1.00 (1.00, 1.00)	1.02 (0.87, 1.20)
LC3	1.03 (0.87,1.20)	0.98 (0.83, 1.15)	1.00 (1.00, 1.00)

TSDI, Townsend Deprivation Index: It is a measure used in social research and public health to assess the level of deprivation or socioeconomic disadvantage within a specific geographical area.

Significant values are in bold.

## Discussion

In this study, we identified three latent classes of lifestyle risk behaviours among UK adults with CVD and their changes over 4 + years. We observed that adults with multiple lifestyle risk behaviours at baseline did not transition to low-risk behaviours over time, which could have significant relevance in daily practices. Gender and age were also significantly associated with the transitions in lifestyle risk behaviours. The odds of transition from LC1 to LC2 was 1.16 times higher with a single-year increase in participants' age. Our findings indicate that individuals in the UK who have CVD should adopt a comprehensive strategy. This strategy involves adhering to recommended alcohol limits, ensuring adequate sleep of 7–8 h per night, consuming at least 5 portions of fruits and vegetables daily, quitting smoking, engaging in regular physical activity, and minimizing prolonged sitting time. These measures collectively address major lifestyle risk factors, supporting improved cardiovascular health and overall quality of life.

The 2019 AHA guideline on the primary prevention of CVD promotes a healthy lifestyle throughout life to reduce CVD incidence (26). Similarly, the 2016 European guidelines on cardiovascular prevention encourage people with established CVD to adopt a healthy lifestyle over time (29). However, in our study, over the 4 + years, UK adults did not show improvement in avoiding multiple lifestyle risk behaviour engagement. Overall, there was an increased engagement in more lifestyle risk behaviours and/or continued practising the same lifestyle risk behaviour over time. This is inconsistent with the NICE guideline on CVD (46), which recommends lifestyle modification for the primary and secondary prevention of CVD. Further research is needed on why people with CVD do not modify their lifestyle risk behaviours over time, and how to promote change.

Clustering and transitioning into multiple lifestyle risk behaviours over time could increase CVD burden and premature mortality. The co-occurrence of multiple risk behaviours has detrimental “synergetic health effects” than would be expected from the added individual effect alone (20–23). A systematic review reported that people with multiple lifestyle risk behaviours were more likely to die from CVD or any cause (25). Similarly, in a large population-based cohort study in Norway, a significant increase in all-cause and cardio-metabolic mortalities was reported, increasing the number of lifestyle risk behaviours (47). This warrants the importance of future interventions targeting multiple lifestyle risk behaviours. People with CVD needs special attention to reduce the risk of disease complication and premature mortality. A meta-analysis of 69 randomized controlled trials reported that education and skills training interventions targeting multiple lifestyle risk behaviours were associated with modest improvements in most lifestyle risk behaviours, such as increased fruit and vegetable intake and physical activity, and reduction in smoking (48).

Transitions in latent classes of lifestyle risk behaviour over the 4 + years differed by gender and age, but not by social deprivation measured in TSDI. The UK male adults showed higher odds of transitioning (2.22) to LC2 (high alcohol intake, and poor fruit and vegetable consumption) over the 4 + years relative staying in LC1 (poor sleep, high alcohol intake, and poor fruit and vegetable consumption). In addition, the odds of moving to LC2 (high alcohol intake and poor fruit and vegetable consumption) relative to staying in LC3 (high alcohol intake) was 4.13 times higher for males than for females. This indicates that high alcohol intake co-occurred mostly with unhealthy eating. This co-occurrence could have a determinantal “synergetic effect” on the daily living and survival of people with CVD. In a systematic review on the clustering of multiple risk behaviours, it was reported that males were at greater risk of engaging in three or more lifestyle risk behaviours (15). Further research is needed to scrutinise gender differences in clustering in multiple lifestyle risk behaviours in people with CVD.

A single-year increase in the age of UK adults at baseline was significantly associated with a 1.16 times increase in the odds of moving to LC1 (high alcohol consumption, poor sleep, and poor fruit and vegetable intake) relative to staying in LC2 (high alcohol consumption, and poor fruit and vegetable intake). There was also a 13.7% less chance of moving to LC3 relative to staying in LC1 with a baseline single-year increase in the age of UK adults. This indicates that ageing is associated with the co-occurrence of multiple lifestyle risk behaviours like poor sleep, high alcohol intake, and poor fruit and vegetable consumption. This could reflect age-related sleep changes—sleep changes as a function of age (49). Ageing is associated with poor sleep: increased awakenings, prolonged nocturnal awakenings, reduced nocturnal sleep duration and decreased deep sleep (50).

Our findings should be considered with the following limitations in mind. Firstly, the UK Biobank collected lifestyle data via self-administered questionnaires, which can introduce inaccuracies and biases. Furthermore, the reliance on volunteer-based participation and absence of real-time monitoring might impact generalizability and introduce potential biases. Secondly, several lifestyle risk behaviours were under-specified in measurement; for instance, sleep data focused solely on quantity, omitting sleep quality. While categorizing smoking as “current smoker” and “not current smoker” simplifies analysis and make the results easier to communicate, it sacrifices detail in transitions between ex-smokers and never smokers, potentially leading to misclassification and less precise outcomes. Such simplification might be less applicable where distinctions matter. Similar challenges arise from the “5-a-day” guideline for fruits and vegetables due to factors like cost, availability, time, cultural preferences, and health constraints, posing consistency challenges. Lastly, applying these findings across settings relies on context due to limited data representativeness. For instance, out of 149,305 participants with physician-diagnosed CVD at baseline, only 10,766 were in the third visit, and just 5,304 had baseline and third visit lifestyle data for our analysis. This highlights data's low representativeness, requiring cautious interpretation of findings.

Overall, despite limitations in measurement, our study has several strengths, including the LTA method, which has greater reliable statistical criteria to identify lifestyle risk behaviour profiles compared to cluster analysis (45). In addition, using a minimum of 4 years for latent transition analysis offers the advantage of identifying stable patterns of lifestyle risk behaviours in individuals with established CVD. This timeframe is valuable as significant behavioural changes are less likely beyond this duration. This study examined and identified lifestyle risk behaviour changes of adults with CVD over time, which has program and policy implications. More research is needed to assess the effects of simultaneous and sequential multiple lifestyle risk behaviour interventions in people with CVD over time.

## Conclusion

UK adults with CVD did not show improvement in lifestyle risk behaviours over time. Either they continued practising the same risk behaviour or engaged in more lifestyle risk behaviours over time. Gender and age were significantly associated with the transitions in lifestyle risk behaviours. Interventions targeting multiple lifestyle risk behaviours either sequentially or concurrently are needed to improve CVD.

## Data Availability

The original contributions presented in the study are included in the article, further inquiries can be directed to the corresponding author.
